# Development and Validation of a Survey on Inclusive Judo: Judo Teachers’ Attitudes Towards Including Participants with Intellectual Developmental Disorders (J-TAID)

**DOI:** 10.3390/sports13010014

**Published:** 2025-01-09

**Authors:** Gaston Descamps, Alain Massart, Terry Rizzo, Viktorija Pečnikar Oblak, Maria João Campos

**Affiliations:** 1 Faculty of Sport and Physical Education, University of Coimbra, 3040-248 Coimbra, Portugal; alainmassart@fcdef.uc.pt (A.M.); mjcampos@fcdef.uc.pt (M.J.C.); 2 Research Unit for Sport and Physical Activity (CIDAF), University of Coimbra, 3040-248 Coimbra, Portugal; 3 Department of Kinesiology, California State University, San Bernardino, CA 92407, USA; trizzo@csusb.edu; 4 Institute of Social Welfare of the Republic of Slovenia, 1000 Ljubljana, Slovenia; viktorija.pecnikar-oblak@irssv.si

**Keywords:** inclusive judo, intellectual developmental disorders, theory of planned behavior, validation, attitudes

## Abstract

This study developed and refined the Judo Teachers’ Attitudes Towards Including Participants with Intellectual Developmental Disorders (J-TAID) survey, addressing the need to assess attitudes, subjective norms, perceived behavioral control, and intention regarding inclusion, and grounded in the Theory of Planned Behavior. The survey, translated into English, Portuguese, French, and Slovenian, was administered to 163 participants in order to assess its reliability and validity using Cronbach’s alpha, Principal Component Analysis (PCA), Confirmatory Factor Analysis (CFA), and Exploratory Factor Analysis (EFA). Internal consistency regarding attitudes, subjective norms, and perceived behavioral Constructs ranged from 0,79 to 0.80, with test–retest reliability improving, demonstrating moderate to strong temporal stability (α = 0.679–0.813). The PCA and CFA identified a robust three-factor structure explaining 74% of the variance, with good model fit (RMSEA = 0.048, CFI = 0.978). Pearson correlations supported the TPB constructs. The refined J-TAID demonstrates validity and reliability for its intended purpose, although the results are still preliminary, and the limitations that were observed suggest a need for further validation.

## 1. Introduction

A recent literature review describes the extensive benefits of judo for individuals with Neurodevelopmental Disorders (NDDs) across the physical, emotional, social, and cognitive domains [1]. Despite these benefits, the literature review underscores a significant gap in the research focusing on judo in inclusive contexts. Previous studies [2,3,4] highlight the benefits of inclusive judo for individuals with Autism Spectrum Disorder (ASD), suggesting that it can promote social interactions, personal growth, and well-being for all participants. However, no known research has focused on the inclusive aspect of judo for individuals with Intellectual Developmental Disorders (IDD), emphasizing the need for such studies. Founded by Jigorô Kanô in 1882, judo integrates ’Ju’ (maximizing potential) and ’Do’ (the journey to mastery). Rooted in Seiryoku zen.yô (efficient energy use) and Jita Kyoei (mutual welfare) [5], judo’s values align with the goals of inclusive education. These philosophies align with UNESCO’s Sustainable Development Goal 4 [6], which champions inclusive and high-quality education for all. Inclusive education is increasingly being recognized as essential. It emphasizes personalized learning that respects diverse abilities, attributes, and aspirations. Sit et al. [7] assert that physical education and physical activity are central to fostering inclusion and can help individuals with disabilities to be included in society. Thus, this study takes inspiration from the pivotal role of inclusion in physical education, as discussed by several scholars [8,9,10,11]. Individuals with IDD face numerous barriers to inclusion in sports, many of which are rooted in their social environment. These barriers include reduced technical proficiency, difficulty interpreting social cues, and a reliance on external support for goal-setting and feedback [12,13]. Moreover, ableist attitudes and social stigma often limit their opportunities for meaningful participation in sports, as highlighted by Sakalidis et al. [14]. In combat sports like judo, instructors can face challenges in balancing group needs and maintaining group cohesion, which can create barriers to effective inclusion for individuals with IDD [15]. These difficulties underline the need for systemic adaptations and targeted training for instructors to overcome those difficulties and to foster equitable participation for all. The critical role of sports teachers in either promoting or restricting participation in inclusive sports underscores the need for targeted training and resources to address these challenges [16,17]. While parallels can be drawn with broader research on inclusive physical education, divergences arise in the application of inclusion principles within combat sports, where interpersonal dynamics and physical engagement play a central role.

Scholars have long emphasized the importance of understanding teachers’ attitudes, as these beliefs significantly influence the inclusion of students with Special Educational Needs and Disabilities (SEND) in physical education [18,19,20,21,22]; however, in the context of judo programs or other combat sports, no similar instruments have been developed. The development of this survey instrument was guided by the Theory of Planned Behavior (TPB). Introduced by Ajzen [23,24,25], the TPB is crucial for understanding, predicting, and influencing behavior. Research on educators’ attitudes toward inclusive physical education often uses this theoretical model [21].

This study describes the development, refinement, and validation of the J-TAID survey, which serves as an initial tool for evaluating judo teachers’ attitudes, subjective norms, perceived behavioral control, and intentions toward inclusion. The iterative nature of this process ensures its alignment with the Theory of Planned Behavior while addressing the methodological challenges. Advice for further validations is also provided.

In some regions, the stigma surrounding disabilities present societal and cultural barriers to inclusion, impacting perceptions of both disability and inclusive practices. These societal attitudes not only marginalize individuals with disabilities but also influence educators’ and coaches’ readiness to implement inclusive programs. Instruments such as the J-TAID are critical for addressing these systemic and interpersonal dynamics, evaluating educators’ beliefs and attitudes, which play a pivotal role in fostering inclusion in sports.

## 2. Methods

The project received ethical approval from the Faculty of Sport Sciences and Physical Education Ethics Committee of the University of Coimbra, with the following reference: CE/FCDEF-UC/00862021.

### 2.1. Qualitative Interviews

#### 2.1.1. Structure of the Interviews

Aligned with the TPB and the work of Ajzen [23,24,25], the J-TAID was developed through qualitative interviews exploring judo teachers’ behavioral, normative, and control beliefs regarding the inclusion of individuals with IDD. This interview process, as part of the same research project, is detailed in the work of Descamps et al. [15], which examined judo teachers’ attitudes toward inclusion by identifying key themes related to both the advantages and challenges of including participants with IDD in judo classes.

#### 2.1.2. Interview’s Conduct

Twenty-one judo teachers from Slovenia (N = 7), Portugal (N = 7), and France/French-Polynesia (N = 7) took part in the interview portion of the survey’s development. Interviews were translated into three languages. Participants included 7 females and 14 males, classified into five experience levels.

#### 2.1.3. Creation of a Vignette

A vignette was created to provide a hypothetical profile of a participant with IDD (gender neutral), aiding in the survey responses. The vignette technique is acknowledged for its capacity to elicit rich insights by presenting scenarios that participants can respond to, facilitating the exploration of reality and values [26,27].

#### 2.1.4. Interview Analysis

Interviews were transcribed, validated, and professionally translated for consistency. Transcriptions were sent back to respondents for verification to ensure validity. Subsequently, data were coded, categorized, and analyzed using the NVivo [28] application. This method of analysis is consistent with that of Young et al. [29], Jamshed [30], and Burnard [31].

### 2.2. Survey Development

#### 2.2.1. Item Creation

The interview analyses revealed the relevant beliefs that could be derived from each group of questions in the interviews. The responses aligned with the TPB construct-guided J-TAID item development [24,25]. The thematic analysis identified salient items from the interview questions. For behavioral beliefs, the analyses of the interviews led to the creation of five strength items: preparation time, which could potentially be more important than usual; a potential reconsideration of teaching skills; the need to create and develop adaptive, inclusive teaching strategies; and the possibility of not having enough time for other participants. For normative beliefs, the analyses of the interviews identified the main referents, who were caregivers of participants with IDD, caregivers of participants without IDD, other judo teachers who included participants with IDD, judo teachers who focused on competition, and the board members of the judo club. For control beliefs, the interview analyses identified five main themes, which were the influence of another instructor, the size of the group (number of participants), the presence of skilled peers, having additional training or education, and whether other participants would understand the nature of the IDD. In the Supplementary Material, the survey’s contents and composition are further explained and illustrated.

#### 2.2.2. Peer Reviewing

Five Adapted Physical Activity (APA) professors peer-reviewed the survey to judge its face and content validity, following the guidelines of Elangovan and Sundaravel [32]. The reviewers agreed the survey had face and content validity and provided valuable advice on how to rephrase items throughout the survey for improved clarity and harmonization. They also offered guidance on terms related to disability, including whether to specify the type of disability at specific points in the survey. Similar consideration was given to the terminology regarding instructing, teaching, and inclusion. The importance of this step is underscored by Taherdoost and Hamed [33] and it aligns with the approaches taken in the evaluation of PEATID-II [34] and PEATID-III [35], proving its critical role in ensuring the validity of the survey.

### 2.3. Pilot Testing

Consistent with the study of Hassan ZA [36], a pilot testing was employed to evaluate the survey’s efficacy and content, gather feedback, and refine the survey based on the responses. The pilot testing played a crucial role in identifying potential issues and deficiencies in the research instruments and protocols before their full implementation at a larger study [37]. Additionally, a primary goal of this research phase was to determine the appropriate effect size for the main research endeavor [38].

#### 2.3.1. Sample

The pilot test included 15 English-speaking judo teachers. Half completed a preprinted questionnaire at a seminar in Ljubljana (Slovenia) and the rest completed the survey electronically. Data collection was anonymous. The sampled group included individuals primarily aged 30–39 years (53.3%, n = 8), with the majority being male participants (66.7%, n = 10), while female participants made up the remaining 33.3% (n = 5). Slovenian participants formed the largest segment (46.7%, n = 7), followed by Portuguese participants (26.7%, n = 4); other participants were from Croatia, Serbia, Russia, and France.

#### 2.3.2. Feedback from Participants

A final open-ended question was included to collect participant feedback and identify issues with the survey [39]. The goal of this step was to ensure that the survey instrument was the best adapted, easily understood, and as clear as possible for the sample of judo instructors; minor adjustments were made after this step. This helped find ambiguous or unclear questions and refine them to be more straightforward and understandable. The goal of the pilot testing was to ensure that the survey’s form was comprehensive and that the questions were well-defined, clearly understood, and presented consistently [37].

#### 2.3.3. Refinement and Design

Other changes were made at the end of the pilot test and during and after the pilot test analysis. The J-TAID was refined, making it more concise and deleting some items that appeared redundant or irrelevant. Pilot test data indicated that it was better and easier to use an online format; therefore, a Google survey was used to ensure efficiency and effectiveness. Some items were better specified to ensure that the respondents fully understood the questions, leading to more exact and reliable responses. For example, the question about time management in the beliefs part was rewritten for clarification.

Sample size estimation. The effect size (d) of the study was calculated as 0.62, based on a hypothesized mean of 3 and a true mean of 3.83; therefore, a minimum sample size of 60 participants was required to detect an effect at a 0.05 or 0.01 significance level. This calculation, using true means for behavioral beliefs and corroborated by Minium [38], ensured an accurate sample size was determined for the study. The formula used is described in the Supplementary Material.

### 2.4. Translations

The peer-reviewed English survey was then translated into Slovenian, Portuguese, and French. Each of the researchers who were part of the project and helped to create this research survey had a different native language (i.e., English, Portuguese, French, and Slovenian). It was, therefore, easier to translate the survey from the English version to the other three languages. The lead investigator, who could speak those four languages, could coordinate the translations with essential help from their colleagues and a professional translator. The method used is described in Valdez [40] as the parallel development approach, which is an alternative strategy for crafting new surveys and questionnaires. Also, consistent with the work of Harkness et al. [41], cultural and language-based feedback regarding the target language(s) and culture(s) was considered during this collaborative process.

### 2.5. Survey Instrument

**Figure 1:**
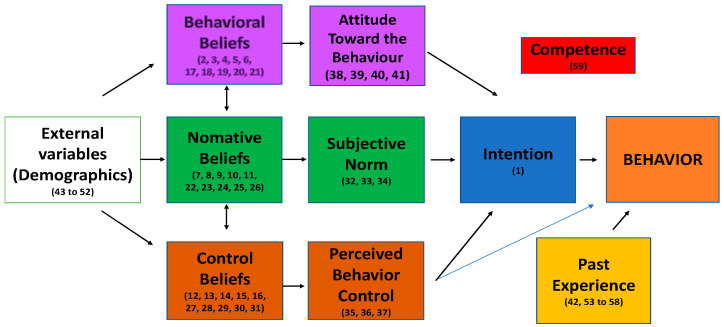
J-TAID, following the TPB [17]. This model includes the addition of external variables from Ajzen and Fishbein [43] regarding the Theory of Reasoned Action.

The J-TAID, as presented in Figure 1, has 41 TPB items: 1 intention item, 10 behavioral beliefs items, 10 normative beliefs items, 10 control beliefs items, 4 items for the construct of Attitude Toward Behavior, 3 for the construct of SN, and 2 for the construct of PBC. One item was about recent Past Behavior, with nine more items referring to the experience of judo teachers. One item was about competence, and the demographic section had a total of nine items. Consistent with the work of Kudláček et al. [42], the scoring system used a 7-point Likert scale. One was high and seven was low for all items. The items included in the J-TAID are presented in the Supplementary Material (Tables S1 and S2).

The TpB specifies how behavioral beliefs influence attitude, normative beliefs influence SN, and control beliefs influence PBC. The TPB predicts how: intention mediates the effects of attitude and SN on behavior; PBC moderates the effect of intention on behavior; and beliefs influence intentions and behavior indirectly through their effects on attitudes, SN, and PBC. The first item of the J-TAID measures the direct measure of intention in a straightforward way: If given the opportunity, I would include Rowan in my judo class. (strongly agree/strongly disagree).

Reports of Participants’ Experiences: At the end of the survey, participants were asked about their experience, such as the number of years they have taught judo. They were also asked if they received any education or training about including participants with IDD. They were asked if they had any experience teaching judo to participants with disabilities, and, if yes, which disabilities, for how many years and in what context (inclusive, separated, individual, or other). They were also asked about the quality of these experiences (very poor, not good, satisfactory, very good, or excellent). The last item of the survey assessed the perceived self-confidence and competence of the judo teachers.

### 2.6. Procedure

The first author sent their survey to judo clubs from France, French Polynesia, the United Kingdom, Ireland, Portugal, and Slovenia. An email list appeared on the websites of each of the country’s judo federations. An invitation via email was sent, including a Google form link to the survey; the first page of the link contained an invitation letter explaining the purpose of the research. If participants agreed to participate, they were asked to sign the agreement showing their “informed, enlightened, and free consent for participation in research studies” This process adheres to the ethical standards outlined in the Declaration of Helsinki [44] and is consistent with the provisions of the Oviedo Convention [45]. How to complete the survey and how the 7-point Likert scale functions were explained. The survey took around 15 min to complete.

### 2.7. Validity and Reliability

A statistical validation of the J-TAID survey was conducted in all four languages—English, Portuguese, French, and Slovenian. This approach was appropriate because the entire survey development process, from the qualitative phase to item creation and translation, was carried out in parallel across these languages to ensure consistency and comparability.

In this study, 163 participants completed the J-TAID. The sample consisted of 76.7% males (N = 125), 22.7% females (N = 37), and 0.6% (N = 1) who did not specify their gender, with a mean age of 48.7 years (SD = 12.3). Participants were from Portugal (N = 44), France (N = 38), Slovenia (N = 32), UK (N = 24), French Polynesia (N = 8), and other countries, and had, on average, about 20 years of experience as judo teachers (SD = 12.6). To ensure the robustness of the validity and reliability of the survey, several statistical tests were conducted, using SPSS and AMOS for data analysis.

Reliability Testing. Cronbach’s alpha [46] was applied to the data to measure internal reliability, indicating how well the items in a construct are correlated to each other. The constructs evaluated included behavioral beliefs, normative beliefs, control beliefs, attitudes, SN, and PBC. High Cronbach’s alpha values suggest that the items within each construct are consistent, with high reliability. The Cronbach’s alpha values for each construct were reported, along with the values if specific items were removed. This helped to identify items that might negatively affect the reliability of the constructs. As recommended by Ajzen [47] and used in earlier research [34,35,48,49,50,51,52,53], Cronbach’s alpha [46] was applied to the data. The benchmark for internal consistency is α ≥ 0.7, which suggests satisfactory reliability.

Test–Retest Reliability. Consistent with Oh et al. [51] when participants completed the J-TAID, participants were asked if they would be willing to take part in a retest. Subsequently, the J-TAID survey was administered a second time, and test–retest correlations were computed. Nineteen participants agreed and completed the survey a second time, allowing for the assessment of test–retest reliability. This follow-up aimed to confirm Cronbach’s alpha for the attitude construct. Additionally, a test–retest Pearson correlation analysis was conducted to further assess reliability, focusing on the attitude construct, measured using four items. The Intraclass Correlation Coefficient (ICC) was also calculated to evaluate the degree of correlation and agreement between measurements. The interval chosen for the test–retest process was 1 to 3 weeks. The threshold for test–retest reliability is ICC > 0.6 for moderate reliability and ICC > 0.8 for excellent reliability.

### 2.8. Factor Analysis

PCA was initially used to explore the factor structure and identify problematic items, EFA was conducted to refine the constructs further and confirm the revised structure, and CFA was subsequently employed on the final model to validate the constructs.

Principal Component Analysis (PCA). Consistent with Kudláček [54], a PCA was conducted to explore the underlying structure of items related to attitudes (ATT), SN, and PBC. This method reduced data dimensionality and assessed communalities, as well as explaining the variance and factor loadings. A revised PCA was performed to enhance the constructs’ validity after identifying and dropping problematic items (SN03 and PBC03).

Principal Axis Factoring (PAF). To corroborate the PCA findings, PAF was employed on the revised item set. This approach further elucidated the relationships between items and their intended constructs, providing additional validation of the survey’s construct structure.

Confirmatory Factor Analysis (CFA). A CFA was employed to confirm the individual constructs, adhering to previously outlined methods [34,35,49]. This analysis ensured that the items aligned with the theoretical constructs and remained distinct from other measures. The internal consistency of the constructs (I, ATB, SN, PBC, Ab, SNb, and PBCb) exceeded the threshold of 0.70 [55], underscoring the reliability of the instrument [56]. The fit indices for CFA are RMSEA < 0.05, CFI/TLI > 0.95.

Exploratory Factor Analysis (EFA). A PAF and Varimax rotation was conducted to explore underlying factors within the dataset. This analysis evaluated initial and extraction communalities, explained the total variance, and rotated factor loadings, enhancing the measurement model.

Pearson Correlation Analysis. Pearson correlation coefficients were computed to assess the relationships between constructs and their respective beliefs. The significant correlations supported the validity of the TPB, indicating that constructs were interrelated as theoretically hypothesized [57]. These analyses were integral to confirming the study’s findings and ensuring robust interpretations [34,53].

## 3. Results

### 3.1. Internal Reliability Measurement

The *p* value for the ICC correlation was *p* < 0.001 for each of the constructs, indicating the reliability of the J-TAID. Table 1 presents the Cronbach’s alpha for each of the constructs. In the Supplementary Material, Table S3 presents the Cronbach’s alpha for deleted items.

The Cronbach’s alpha was calculated for each of the constructs after removing irrelevant items to improve internal consistency. For behavioral beliefs, three items (BB01, BB02, and BB03) were found to negatively impact reliability, with their removal improving the alpha from 0.52 to 0.62. The normative beliefs scale had one problematic item (NB04), and its removal increased the alpha coefficient from 0.74 to 0.78. The control beliefs scale was relatively consistent, with an overall alpha of 0.62, although items such as CB01 (alpha = 0.62) and CB04 (alpha = 0.64) may require refinement to improve the scale further. The subjective norms (SN) scale was impacted by one item (SN03), and had a high alpha of 0.79 when this item was removed. In the perceived behavioral control (PBC) construct, after removing item PBC03, an alpha of 0.80 was obtained. The Attitude construct showed a Cronbach’s alpha of 0.79.

### 3.2. Test–Retest Reliability

Cronbach’s alpha values for the test administration indicated moderate internal consistency (α = 0.68. After the retest, the Cronbach’s alpha values demonstrated excellent internal consistency (α = 0.813). The ICC for single measures during the test’s administration indicated low to moderate reliability (ICC = 0.346; 95% CI = 0.120 to 0.610; *p*-value < 0.001), whereas the average measures ICC indicated good reliability (ICC = 0.679; 95% CI = 0.354 to 0.862; *p*-value <0.001). For the retest, the ICC for single measures improved to moderate reliability (ICC = 0.520; 95% CI = 0.293 to 0.741; *p*-value < 0.001) and the average measures ICC indicated excellent reliability (ICC = 0.813; 95% CI = 0.623 to 0.920; *p*-value < 0.001). The Pearson Correlation analysis revealed a significant positive correlation between the test and retest scores (r = 0.736, N = 19, *p* < 0.001), indicating the strong reliability of the instrument. Importantly, the *p*-values were consistently less than 0.001 for all reliability metrics.

### 3.3. Principal Component Analysis (PCA)

The initial communalities were 1.00, as expected, with extraction communalities ranging from 0.46 (PBC03) to 0.88 (SN03). Three components had eigenvalues greater than 1, explaining 64.6% of the variance (39.2%, 15.3%, and 10.2%, respectively). However, SN03 appeared irrelevant due to its lack of correlation with the other items, and PBC03 showed low communalities and factor loadings. Varimax rotation clarified the structure: Component 1 included the PBC01, PBC02, and ATT items (which also loaded on Component 2). Component 2 included SN01 and SN02, while Component 3 was dominated by SN03. A revised PCA excluded SN03 and PBC03 to improve the model. The updated communalities ranged from 0.62 to 0.87, and the three components explained 74.0% of the variance (46.6%, 17.5%, and 9.9%, respectively). The Varimax rotation results showed the following: Component 1 included PBC01, PBC02, and ATT items; Component 2 included SN01 and SN02; Component 3 primarily included ATT items. This revised structure better aligns with the theoretical expectations.

### 3.4. Factor Analysis

Using Principal Axis Factoring (PAF), extraction communalities ranged from 0.38 (ATT02) to 0.72 (PBC01), showing that the factors explained most of the variance in the items. The first three factors accounted for 41.7%, 13.5%, and 4.7% of the variance, respectively. The rotated solution clarified the structure, consistent with the PCA results, as presented in Table 2.

### 3.5. Confirmatory Factor Analysis (CFA)

The CFA confirmed the adequacy of the proposed measurement model. The chi-square statistic (χ^2^ = 43.876, df = 32) was significant but is known to be sensitive to sample size. Additional fit indices indicated good model fit: RMSEA = 0.048 (excellent), CFI = 0.978, TLI = 0.968 (both above 0.95), and SRMR = 0.062 (<0.08). The AIC (4142.37) and BIC (4213.526) supported model comparison, with lower values indicating better fit. Factor loadings and error variances are detailed in Table 3.

CFA with computed beliefs and reduced beliefs. The initial CFA with computed beliefs showed a moderate fit (χ^2^ = 212.208; RMSEA = 0.094; SRMR = 0.107; CFI = 0.709; TLI = 0.649; AIC = 6680.905; BIC = 6782.999), suggesting room for improvement. A revised CFA with reduced computed beliefs improved the fit, with χ^2^ = 169.046, CFI = 0.740, and TLI = 0.673, and reduced the AIC (5750.185) and BIC (5839.904). Despite slight increases in RMSEA (0.103) and SRMR (0.111), the model quality improved overall. The correlations between factors also changed: behavior and normative beliefs strengthened (from 0.02529 to 0.07317), behavior and control beliefs showed minor growth (from 0.00132 to 0.00287), and normative and control beliefs remained nearly unchanged (from −0.00449 to −0.00448). Factor loadings before and after the reduction, as shown in Table S4 (Supplementary Material), highlight these improvements. The other covariances remained stable, indicating that the factors are independent, as presented in Table S5 (Supplementary Material).

### 3.6. EFA Results of Reduced Computed Beliefs

The EFA was conducted using PAF and Varimax rotation; the results are summarized in the Table 4 here and in the Table S6 (Supplementary Material). The factor analysis identified three factors that explain a cumulative variance of about 39.9%.

### 3.7. Pearson Correlations

Beliefs and their constructs. Attitudes correlated positively with behavioral beliefs (r = 0.59, *p* < 0.001), indicating a strong linear relationship. Subjective Norms (SN) correlated moderately with normative beliefs (r = 0.44, *p* < 0.001), and Perceived Behavioral Control (PBC) correlated negatively with control beliefs (r = −0.26, *p* < 0.001).

Correlation between Constructs. Attitudes correlated positively with SN (r = 0.41, *p* < 0.001) and PBC (r = 0.54, *p* < 0.001). A weaker but significant positive correlation was observed between SN and PBC (r = 0.198, *p* = 0.012).

### 3.8. Correlation Between Beliefs

Behavioral beliefs correlated positively with normative beliefs (r = 0.202, *p* = 0.010), while correlations with control beliefs were insignificant (r = 0.09, *p* = 0.255). Similarly, normative beliefs showed no significant correlation with control beliefs (r = −0.028, *p* = 0.718).

## 4. Discussion

This study was designed to develop a valid survey (J-TAID) to assess and evaluate judo teachers’ attitudes, SN, PBC, and intentions regarding the inclusion of individuals with IDDs in judo classes, anchored in the robust framework of the TPB [23,24,25]. The J-TAID aligns with the broader field of inclusive physical education/activity studies and studies assessing teachers’ attitudes [21], as their role and attitude are critical in shaping an inclusive climate in sports or physical education classes. The methodology employed in the J-TAID’s development—encompassing qualitative interviews and rigorous translation processes—ensures its cultural relevance and international applicability.

### 4.1. Internal Reliability Measurement

Cronbach’s alpha coefficients indicate varying reliability levels. Constructs like Attitudes and normative beliefs were very satisfactory, while SN and PBC showed good reliability too. These findings align with previous studies [34,35,48,49,50,51,52,53]. For the behavioral and control beliefs, these constructs showed moderate reliability, which could be improved by reviewing the content of each item to ensure better alignment. The increase in alpha for SN and PBC upon the removal of the third items suggests that these items may negatively impact the overall reliability of the construct. The reliability metrics consistently showed strong statistical significance (*p* < 0.001), underscoring the instrument’s robustness.

Although the Cronbach’s alpha values for behavioral and control beliefs composites fell below α = 0.7, this alpha value is not critical for the belief composites, as inherent variability is expected in the exploratory phases and accessible beliefs may inherently exhibit variability. The constructs of attitudes (α = 0.79) and subjective norms (α = 0.79 after refinement), as well as that of perceived behavioral control (α = 0.80 after refinement) exceeded the threshold, validating the scale’s reliability for key constructs.

### 4.2. Test–Retest Reliability

The test–retest reliability analysis revealed increased the Cronbach’s alpha and ICC, indicating improvements in the internal consistency and stability of the attitude construct. The significant Pearson correlations support this reliability, likely due to the participants’ familiarity increasing or their attitudes becoming more stable over time. We hypothesize that participant familiarity with the survey contributed to these improvements, as repeated exposure allowed for greater clarity and understanding of the items. These findings indicate preliminary indicators of reliability.

### 4.3. Convergent and Discriminant Validity

The initial PCA revealed issues with both third items of the SN and PBC constructs. Their removal improved the model. The SN and PBC constructs are now measured using only two items each, which shortens the J-TAID, making it easier for participants to complete. The overall model aligns well with the theoretical expectations and improves the stability and interpretability of the constructs.

### 4.4. Factor Analysis

The CFA validated the measurement model, with fit indices (RMSEA, CFI, TLI, SRMR) showing good alignment between the hypothesized and observed data. Items with low factor loadings or high error variances, such as the third items of SN and PBC, required revision. These refinements improved the model fit and confirmed the validity of the construct. The updated model showed better fit indices, reduced AIC/BIC values, and strong, consistent factor loadings. Correlations between behavior and normative beliefs increased, reflecting a stronger relationship. The EFA revealed a three-factor solution with improved communalities and well-defined constructs, suggesting that reducing computed beliefs enhances fit while preserving measurement reliability.

### 4.5. Construct Validity

The CFA results showed strong factor loadings for most items, indicating that they are good indicators of latent constructs. The intercorrelations among the TPB constructs (attitudes, SN, and PBC) revealed moderate to strong positive correlations, aligning with the theoretical and empirical expectations. A second CFA, using reduced computed beliefs, showed consistent factor loadings across models, confirming the stable relationships between the indicators and latent constructs. However, the covariances varied slightly, reflecting shifts in the relationships between latent variables. Overall, the updated survey fits better according to most criteria but could benefit from further refinement to improve fit indices like RMSEA and SRMR. Further investigation into the latent variable relationships will help enhance the survey.

### 4.6. Pearson Correlations

The Pearson correlation analysis supports the validity of the Theory of Planned Behavior (TPB), showing significant relationships among the constructs and their respective beliefs (*p* < 0.001). Favorable attitudes correlate with favorable behavioral beliefs and a sense of control (PBC) regarding the inclusion of participants with IDD. PBC is linked to a lower perceived need for facilitative conditions. The correlations between these constructs reveal that favorable attitudes are associated with compliance with SN, having control (PBC), and greater ease in including participants with IDD in judo. Attitudes positively relate to SN and PBC, with a stronger link to PBC, while SN and PBC are positively but weakly correlated. Higher behavioral beliefs align with compliance with SN beliefs. These findings confirm the interrelationships of the TPB constructs, as hypothesized [57].

### 4.7. Practical Implications

Favorable attitudes toward including participants with IDD are associated with positive behavioral beliefs and greater PBC. These attitudes correlate with compliance with social norms (SN), suggesting that positive attitudes enhance confidence and conformity with supportive norms. While SN and PBC are interrelated, attitudes play a more significant role. The findings imply that fostering positive attitudes and strong social norms can improve perceived control and effectiveness in inclusive practices, improving outcomes. The qualitative component of the same research project [15] provides further insights. Judo teachers expressed generally positive attitudes toward inclusion, highlighting both pedagogical benefits and the challenges associated with teaching individuals with IDD. Teachers noted that inclusion fosters empathy, patience, and creativity, which aligns with the positive attitude scores obtained in the quantitative analysis. However, the qualitative data also revealed concerns about time constraints, group cohesion, and increased cognitive demands, which correspond with the moderate reliability of the control beliefs in the quantitative results. Both parts of this research project reinforce the need for targeted training and resources to support judo teachers in overcoming the practical obstacles they associate with inclusion.

### 4.8. Limitations of the Study

The reliance on Cronbach’s alpha to assess internal consistency is standard practice, yet it may not adequately address the multidimensionality of the constructs. The use of PCA and EFA, while useful for initial validation, has limitations in terms of sample size adequacy and the assumptions underlying these methods. The CFA that was performed indicated the J-TAID is a respectable survey. However, a larger sample size might better confirm the robustness of the survey using TPB as its model. We acknowledge that the reuse of data for PCA and EFA introduces a risk of overfitting, and the findings should be interpreted as exploratory. Future research should validate the scale using independent datasets for confirmatory and exploratory analyses. Also, the beliefs composites could benefit from refinements, including revisiting the wording of items and increasing item specificity to enhance internal consistency in the beliefs constructs.

Additionally, this multilingual survey, despite rigorous translation efforts, may still encounter issues with cultural nuances that affect the consistency and validity of the responses.

### 4.9. Suggestions for Future Studies

Future studies should employ larger, more diverse samples and consider using advanced statistical techniques like Item Response Theory (IRT) for better validation. Also, future research should alter the vignettes used and describe a participant with another disability to expand the research on inclusive judo to different populations. It would also be interesting to conduct further interviews with potential participants to explore various influences on their beliefs and subsequent constructs. Regarding the test–retest process, we propose that future studies extend the research interval and involve more diverse participants to confirm these results.

## 5. Conclusions

The refined J-TAID is a valid and reliable tool for assessing inclusion attitudes among judo teachers. While preliminary, these findings provide a foundation for further research and iterative validation efforts. The J-TAID could benefit from further refinement and validation. Simplifying the scale by reducing the number of items and refining the terminology may enhance its usability and psychometric properties.

Iterative item revision and model refinement improved the instrument’s alignment with the theoretical expectations and psychometric properties. J-TAID, available in the English, Portuguese, French, and Slovenian languages, is a valuable tool for assessing the state of inclusion in judo from the judo teachers’ point of view. The J-TAID could also be useful for the development of programs to target judo teachers, measuring pre–post changes following an awareness intervention.

## Figures and Tables

**Table 1:** Cronbach’s alpha score for each of the constructs.

Construct	Number of Items	Cronbach’s Alpha
Behavioral Beliefs	10	0.59
Normative Beliefs	10	0.76
Control Beliefs	10	0.63
Attitudes	4	0.79
SN	3	0.52 (initial), 0.79 (after refinement)
PBC	3	0.68 (initial), 0.80 (after refinement)

**Table 2:** Rotated Factor Matrix.

	Factor		
	1	2	3
SN01	0.17	**0.75**	0.35
SN02	−0.04	**0.81**	0.14
PBC01	**0.76**	0.07	0.37
PBC02	**0.76**	0.02	0.24
ATT01	0.24	0.14	**0.63**
ATT02	0.15	0.25	**0.54**
ATT03	0.26	0.15	**0.63**
ATT04	0.36	0.25	**0.71**

Factor 1 included PBC01 and PBC02, Factor 2 included SN01 and SN02, and Factor 3 included ATT01, ATT02, ATT03, and ATT04.

**Table 3:** Factor loadings and error variances.

Path	Estimate	Standard Error	Lambda (λ)	Error (e)	Relationship Description
ATT → ATT02	0.857	0.130	0.85656	0.66107	Strong relationship
PBC → PBC02	0.842	0.095	0.84195	0.44090	Strong relationship
SN → SN02	0.645	0.125	0.64484	0.57998	Moderate relationship
SN → SN03	0.106	0.102	0.10550	0.98876	Poor indicator
ATT → ATT03	1.010	0.133	1.01002	0.52874	Unusually high loading
ATT → ATT04	1.257	0.140	1.25679	0.27033	Unusually high loading
PBC → PBC03	0.452	0.096	0.45241	0.83857	Weaker relationship
PBC → PBC01	0.211	0.073	Not Applicable	0.21128	Low error variance
ATT → ATT01	Not Applicable	Not Applicable	Not Applicable	0.53805	Factor loading not applicable

**Table 4:** Variance explained by factors.

Factor	Initial Eigenvalues			Extraction Sums of Squared Loadings			Rotation Sums of Squared Loadings		
	Total	% Variance	Cumulative %	Total	% Variance	Cumulative %	Total	% Variance	Cumulative %
1	2.81	25.5%	25.5%	2.32	21.1%	21.1%	2.07	18.8%	18.8%
2	2.06	18.7%	44.2%	1.43	13.0%	34.0%	1.30	11.8%	30.6%
3	1.26	11.4%	55.6%	0.64	5.9%	39.9%	1.02	9.2%	39.9%

Factor loadings indicate the correlation of each variable with the factor. Table 4 presents the loadings after rotation for both solutions.

## Data Availability

All data generated or analyzed during this study are included in the article as Table(s).

## Supplementary Materials

The supporting information can be downloaded at: https://www.mdpi.com/article/10.3390/sports13010014/s1, including Table S1: Type of beliefs and Illustrative Items; Table S2: Type of Constructs and Illustrative Items; Table S3: Cronbach Alpha with items deleted; Table S4: Comparison of CFA Model Fit and Factor Loadings Before and After Reduction of Computed Beliefs; Table S5: Covariance Changes Between Belief Factors Before and After Reduction of Computed Beliefs; Table S6: 3-Factor Solution (Rotated).
