# Supplementary material for: Development and Validation of a Survey on Inclusive Judo: Judo Teachers’ Attitudes Towards Including Participants with Intellectual Developmental Disorders (J-TAID)
Source: Sports (Basel). 2025 Jan 9;13(1):14. doi: 10.3390/sports13010014 (PMC11769555; doi:10.3390/sports13010014)
Supplement: Supplementary file 1 [file sports-13-00014-s001.zip › sports-3386126-supplementary.pdf]

Supplementary A : content of the survey

Table S1. Type of beliefs and Illustrative Items

**Behavioral Belief**
Belief Strength<br>Including Rowan will require me to be more creative by developing adaptive, inclusive teaching strategies.<br>*Strongly agree/Strongly disagree*<br><br>Belief Outcome Evaluation<br>For me to develop adaptive inclusive teaching strategies to accommodate Rowan is. *Extremely good/Extremely bad*
**Normative Belief**
Normative referents<br>Caregivers of participants without disabilities think I should include Rowan.<br>*Strongly agree/Strongly disagree*<br><br>Motivation to Comply<br>Generally speaking, I would do what the:<br>caregivers of participants without disabilities think I should do.<br>*Definitely yes/Definitely no*
**Control Beliefs**
Control Belief Strength<br>I can include Rowan without the help of another instructor.<br>*Strongly agree/strongly disagree*<br><br>Control Belief Power<br>The presence of another instructor on the mat will facilitate Rowan's inclusion.<br>*Strongly agree/Strongly disagree*

As presented in Table 1, each of the beliefs is related to a statement about its outcome. The strength of each accessible belief (b) is multiplied by the subjective evaluation (e) of the outcome or experience and the resulting products are summed. In the TPB, beliefs are summed to produce composites of behavioral, normative, and control beliefs (Σbiei, Σnimi, Σcipi). For example, behavioral beliefs are theorized to produce a positive or negative attitude toward behavior in their aggregate. Specifically, the positive or negative valence of each expected outcome or experience contributes to the overall attitude in direct proportion to the subjective probability that the behavior will produce the outcome or experience in question. In their respective aggregates, behavioral beliefs produce a favorable or unfavorable attitude toward including Rowan in judo classes. Attitude ∝ ∑=biei. A person's attitude (ATT)is expected to be directly proportional (/) to this composite belief index. Six items from the belief's components (BB5, BB6, BB10, NB4, CB1, and CB4) are considered reversals by their phrasing. When entered in the SPSS program, they were computed to ensure consistency and accuracy in the analysis by reversing the scoring of these items before further statistical procedures were conducted; lower scores were considered more favorable than higher scores.

Table S2. Type of Constructs and Illustrative Items

**Attitude Toward the Behavior**
Including Rowan with participants without disabilities is:<br>*Extremely good/Extremely bad*
**SN**
Most of the people important to me think I should include Rowan.

Strongly agree/Strongly disagree
**PBC**
To what extent do you feel confident you have the necessary resources and support to include Rowan. effectively?<br>*Confident/Not confident*

In the demographic part, the participants were asked about their gender, age, nationality, country of residence, professional status, judo belt and highest degree earned.

Supplementary B : Sample size estimation.

Sample size estimation. The sample size estimation for the current study involves the calculation of the effect size (d) for a particular group mean. The formula (Minium, E., 1978) used is:

$$d = \frac{\text{true mean} - \text{hypothesized mean}}{\text{Sd}}$$

grand hypothesized mean : 3

grand true mean : 3,83

d = (3,83 – 3) / 1,33 = 0,62

For the specific scenario in this study, where the grand hypothesized mean is 3, and the grand true mean is 3.83, the calculated effect size (d) is 0.62. This value indicates (on a table from Minium, 1978, page 374) that, a minimum sample size of 60 participants is necessary for the study to measure any existing effect if one exists. Notably, Minium (1978) further corroborates this requirement, suggesting that with a mean of 3.83, a sample size ranging from 55 to 70 participants is necessary to detect an effect at the 0.05 or 0.01 significance level. The true means for behavioral beliefs were used in this calculation, with the lead investigator setting the hypothesized mean to ensure a comprehensive and accurate sample size determination for the study.

Supplementary *C*: . *Cronbach Alpha with items deleted*

Table S3. Cronbach Alpha with items deleted

**Construct**	**Item**	**Cronbach's Alpha if Item Deleted**
Behavioral Beliefs	BB01	0.62
	BB02	0.59
	BB03	0.52
	BB04	0.50
	BB05	0.59

	BB06	0.61
	BB07	0.54
	BB08	0.55
	BB09	0.52
	BB10	0.60
Normative Beliefs	NB01	0.76
	NB02	0.75
	NB03	0.74
	NB04	0.78
	NB05	0.76
	NB06	0.71
	NB07	0.72
	NB08	0.72
	NB09	0.72
	NB10	0.72
Control Beliefs	CB01	0.62
	CB02	0.55
	CB03	0.62
	CB04	0.64
	CB05	0.61
	CB06	0.57
	CB07	0.58
	CB08	0.62
	CB09	0.58
	CB10	0.61
SN	SN01	0.36
	SN02	0.17

	SN03	0.79
PBC	PBC01	0.45
	PBC02	0.50
	PBC03	0.80
Attitudes	ATT01	0.75
	ATT02	0.78
	ATT03	0.74
	ATT04	0.70

Supplementary D : . Comparison of CFA Model Fit and Factor Loadings Before and After Reduction of Computed Beliefs

Table S4. Comparison of CFA Model Fit and Factor Loadings Before and After Reduction of Computed Beliefs

Factor	Item	Before (Estimate)	Update After (Estimate)	Update Std. Error
Behavior Beliefs (BB)	BB01C	0.987	-	0.109
	BB02C	0.878	0.88549	0.101
	BB03C	-0.111	-0.18452	0.277
	BB04C	0.905	0.91387	0.105
	BB05C	0.942	0.94245	0.104
Normative Beliefs (NB)	NB01C	0.416	0.41460	0.066
	NB02C	0.259	0.26162	0.067
	NB03C	0.688	0.68792	0.086
	NB04C	0.948	0.94727	0.106
	NB05C	0.791	-	0.092
Control Beliefs (CB)	CB01C	0.596	0.60225	0.143
	CB02C	0.625	0.61372	0.136
	CB03C	0.999	-	0.111
	CB04C	0.818	0.81826	0.111
	CB05C	0.958	0.96120	0.111

TableS5. Covariance Changes Between Belief Factors Before and After Reduction of Computed Beliefs

Beliefs Pair	Before (Correlation)	Update After (Correlation)	Update Std. Error
Behavior Beliefs and Normative Beliefs	0.02529	0.07317	0.02
Behavior Beliefs and Control Beliefs	0.00132	0.00287	0.003
Normative Beliefs and Control Beliefs	-0.00449	-0.00448	0.011

Supplementary E: 3-Factor Solution (Rotated)

Table S6. 3-Factor Solution (Rotated)

Variable	Factor 1	Factor 2	Factor 3
BB02C		0.64	
BB03C		0.60	
BB04C		0.53	
NB01C	0.75		
NB02C	0.78		
NB03C	0.63		
NB04C	0.32		
NB05C	0.46		
CB01C			0.60
CB02C			0.65
CB04C			0.39
